# A rare case of urosymphyseal fistula following robot-assisted radical prostatectomy

**DOI:** 10.1016/j.eucr.2025.103066

**Published:** 2025-05-12

**Authors:** Viliam Kubas, Terézia Hrubá, Vladimír Baláž, Jozef Babeľa, Natália Farraová, Boris Hudec, Jana Poláková Mištinová

**Affiliations:** a Urology Clinic, F. D. Roosevelt University Hospital, Námestie L. Svobodu 1, 97517, Banská Bystrica, Slovakia; b Jessenius a.s., Štefánikova 72/46A, 94901, Nitra, Slovakia; c Radiology Clinic, Comenius University, Limbová 5, 83305, Bratislava, Slovakia

**Keywords:** Urosymphyseal fistula, Prostate cancer, Robotic-assisted radical prostatectomy, MRI, Case report

## Abstract

Urosymphyseal fistula (USF) is a rare but serious complication following robot-assisted radical prostatectomy (RARP), frequently associated with osteomyelitis of the pubic symphysis and causing significant morbidity. We present a case of a 66-year-old patient who developed USF with osteomyelitis after RARP, characterized by persistent pelvic pain and urinary symptoms. MRI was crucial in diagnosing the fistula and osteomyelitis, guiding a successful multidisciplinary intervention, including robotic-assisted surgery. Early diagnosis, advanced imaging modalities, and collaborative surgical management remain essential for optimal patient outcomes. Continued advancements in diagnostic and therapeutic strategies are necessary for effective management of USF.

## Highlights

• Urosymphyseal fistula is a rare but serious complication following robot-assisted radical prostatectomy, often leading to significant patient morbidity.
• Early diagnosis through advanced imaging techniques like MRI is crucial for timely intervention.
• A multidisciplinary approach, including robotic surgery and infection management, plays a key role in improving patient outcomes.
• This case highlights the importance of awareness, early detection, and comprehensive treatment to enhance recovery and quality of life for affected patients.

## 1 Introduction

Urosymphyseal fistula (USF) with osteomyelitis of the symphysis pubis is a rare but increasingly recognized complication of prostate cancer treatment, particularly surgery or radiotherapy. There are several hypotheses about its pathogenesis, but the condition is still not well understood. The phenomenon manifests as an abnormal connection between the urinary tract and the pubic symphysis, leading to significant morbidity. The prevalence of USF may be underestimated because its non-specific symptoms, including pelvic pain, limited mobility and recurrent infections, may delay diagnosis. Recent studies have highlighted the increasing awareness and improved detection of USF through advanced imaging techniques such as magnetic resonance imaging (MRI) and multidisciplinary care.^1^

## 2 Case report

**Fig. 1:**
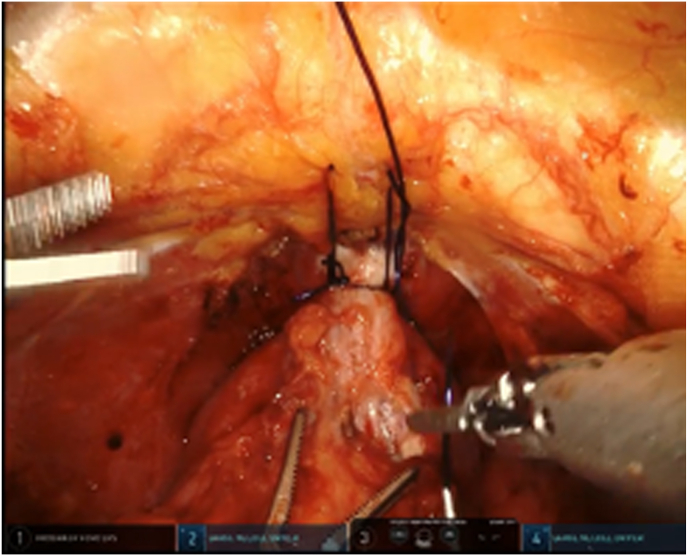
An anterior approach with periurethral suspension suture.

A 66-year-old patient with an elevated PSA level of 14.64 ng/ml and urinary retention requiring permanent catheterization was diagnosed with prostate adenocarcinoma (Gleason 7, ISUP grade 2). Metastatic work-up including a PSMA PET scan was negative. The patient underwent transperitoneal robot-assisted radical prostatectomy without pelvic lymph node dissection. An anterior approach was utilized, and a periurethral suspension suture made of absorbable material was placed intraoperatively to minimize postoperative urinary incontinence (Fig. 1).

During surgery, multiple small calcifications associated with the patient's urinary catheter were identified and aspirated from the bladder. After completion of the anastomosis, a leak test was performed by instilling 300 ml of fluid into the bladder. A closed-suction Redon drain was placed in the retroperitoneal space. The patient was discharged on postoperative day 2, and the indwelling Foley catheter was removed on postoperative day 7. We advised the patient to repeat his PSA level two months later. Final pathology confirmed localized prostate cancer classified as pT3a, pN0, pMX, with positive surgical margins. Adjuvant radiotherapy was not administered.

**Fig. 2:**
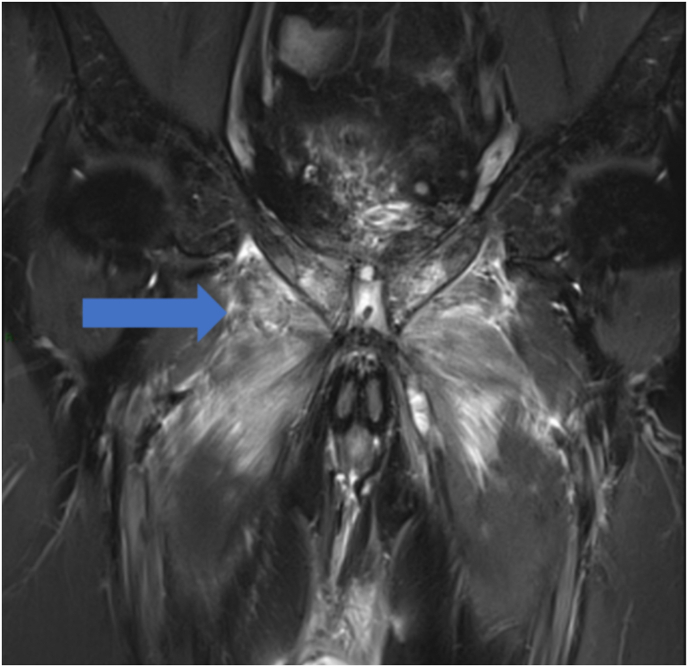
MRI, a, T2 FS-weighted coronal MRI sequence showing symmetrical edema involving the pectineus, obturator and adductor muscles.

**Fig. 3:**
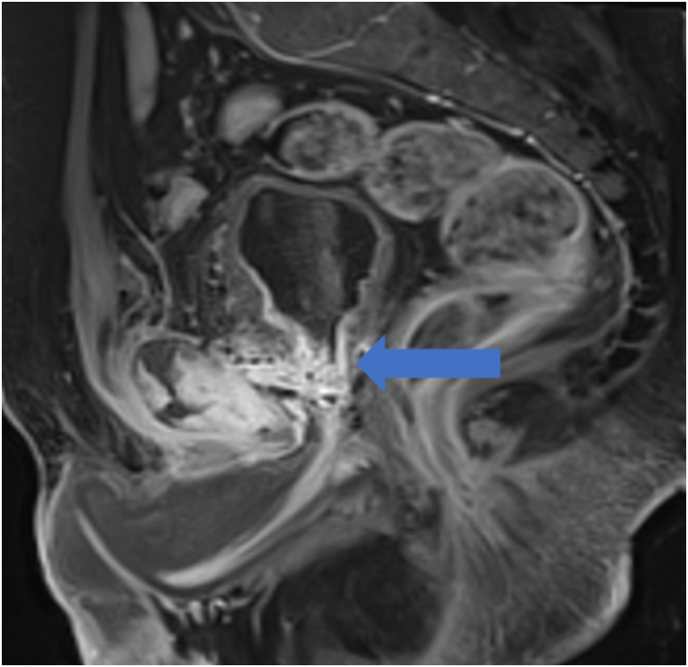
Post-contrast SAG T1-weighted MRI sequence showing communication between the bladder and symphysis with patological enhancement and abscess formation.

Approximately one month after surgery, the patient began experiencing persistent groin pain, subfebrile temperatures, impaired mobility, and difficulty walking. Ultrasonographic examination performed at that time revealed no suspicious findings, and cystoscopy was not indicated. The first postoperative urine cultures remained sterile, and initial antibiotic therapy with ciprofloxacin did not resolve the problems. The patients's symptoms continued to worsen. Approximately five months postoperatively, an MRI revealed a urosymphyseal fistula, extensive abscess formation, pronounced muscular edema involving the adductor and obturator muscles, and bilateral osteitis with early osteomyelitis of the pubic bone (Fig. 2, Fig. 3).

**Fig. 4:**
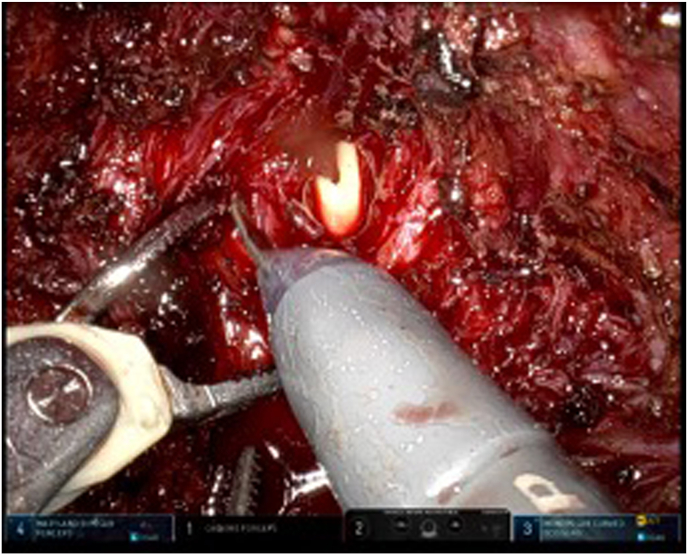
Bladder opening after fistula excision.

**Fig. 5:**
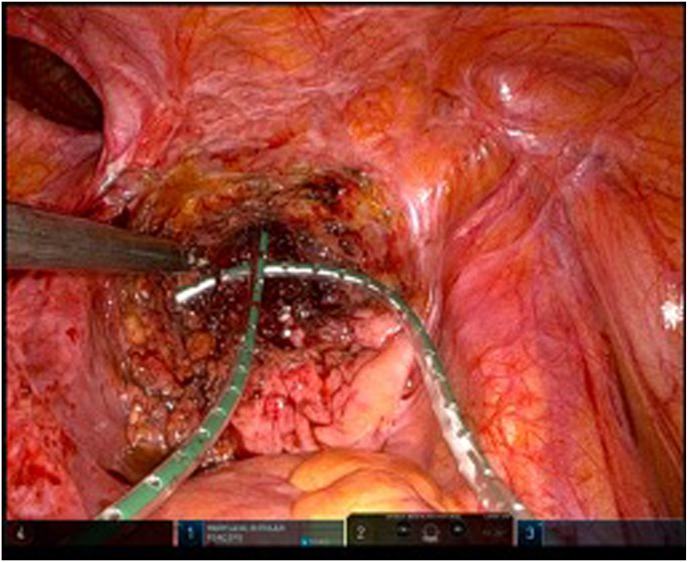
Final state photograph after bladder closure and drainage.

Shortly after the MRI was performed, a multidisciplinary seminar was held. At the multidisciplinary seminar, a decision was made to proceed with a robotic-assisted surgical revision, which included fistula excision, extensive abscess drainage, and thorough pubic bone debridement (Fig. 4, Fig. 5).

Intraoperatively, samples were taken for culture, which revealed a *Proteus mirabilis* infection. Postoperative management involved targeted combination antibiotic therapy and supportive care, leading to gradual symptomatic improvement. The patient demonstrated steady recovery, resuming mobility, experiencing reduced pain, and regaining urinary continence. Continued antibiotic therapy and close monitoring were planned for ongoing management.

## 3 Discussion

Urosymphyseal fistula following robotic radical prostatectomy is a rare but serious complication of unclear aetiology. While it is often associated with pelvic radiotherapy, USF can also occur after robotic prostate surgery due to factors such as infection, tissue necrosis or extensive pelvic dissection. Osteomyelitis of the pubic symphysis, a related condition, is extremely rare, accounting for less than 1 % of all osteomyelitis cases.^2^

A potential cause of osteomyelitis is the suturing of the dorsal venous complex (DVC) during RARP using V-lock suture needles anchored at the level of the pubic bone. This technique has been hypothesised to carry a risk of infection or trauma to the pubic bone leading to osteomyelitis.^3^ In addition, long-term catheterization combined with the formation of small calcifications may create an environment conducive to infection. Removal of these calcifications during the perioperative period may facilitate the spread of infection. Although urine leak and infected urinoma could represent potential causes, intraoperative testing of the anastomosis and the absence of any signs of urinary leakage made them unlikely.

A study from the Cleveland Clinic highlighted that most cases of USF require surgical intervention.^4^ Treatment typically involves bladder reconstruction, pubic bone debridement and in some cases urinary diversion. Postoperative outcomes are generally favourable, with significant pain relief and improved quality of life reported by patients.

Early diagnosis is essential for effective management of USF. Pelvic MRI remains the diagnostic tool of choice, allowing detailed visualisation of fistulas and associated complications. A multidisciplinary approach is essential for successful management, combining surgical expertise, infection control and long-term follow-up.

In conclusion, this case illustrates the diagnostic and therapeutic complexities of managing USF after RARP. Early recognition and comprehensive management can significantly improve outcomes for affected patients. Further research is needed to establish evidence-based guidelines for the prevention, diagnosis and treatment of USF.

## CRediT authorship contribution statement

**Viliam Kubas:** Investigation, Methodology, Writing – original draft. **Terézia Hrubá:** Data curation, Visualization, Writing – original draft. **Vladimír Baláž:** Conceptualization, Methodology. **Jozef Babeľa:** Conceptualization, Methodology. **Natália Farraová:** Data curation, Visualization. **Boris Hudec:** Supervision, Validation. **Jana Poláková Mištinová:** Writing – review & editing.

## Ethics approval statement

The ethical approval was not required.

Written informed consent has been obtained from the patient.

## Funding

This research received no external funding.

## Conflict of interest

The authors declare no conflict of interest.
